# Identification of a New Endo-β-1,4-xylanase Prospected from the Microbiota of the Termite *Heterotermes tenuis*

**DOI:** 10.3390/microorganisms10050906

**Published:** 2022-04-26

**Authors:** Olinda S. A. Alcobaça, Emeline B. Campanini, Iara Ciancaglini, Sâmara V. Rocha, Iran Malavazi, Caio C. M. Freire, Francis M. F. Nunes, Andrea S. C. Fuentes, Anderson F. Cunha

**Affiliations:** 1 Department of Genetics and Evolution, Federal University of São Carlos, Sao Carlos 13565-905, Brazil; olyathaiden@gmail.com (O.S.A.A.); emelinebc@gmail.com (E.B.C.); samara.vieira@hotmail.com (S.V.R.); imalavazi@ufscar.br (I.M.); caiomfreire@gmail.com (C.C.M.F.); francis.nunes@ufscar.br (F.M.F.N.); andreasc@ufscar.br (A.S.C.F.); 2 Brazilian Biorenewables National Laboratory, Brazilian Center of Research in Energy and Materials, Campinas 13083-100, Brazil; i.ciancaglini@hotmail.com

**Keywords:** *Heterotermes tenuis*, meta-transcriptome, symbiotic protist, hemicellulase, xylanase, second-generation bioethanol

## Abstract

Xylanases are hemicellulases that break down xylan to soluble pentoses. They are used for industrial purposes, such as paper whitening, beverage clarification, and biofuel production. The second-generation bioethanol production is hindered by the enzymatic hydrolysis step of the lignocellulosic biomass, due to the complex arrangement established among its constituents. Xylanases can potentially increase the production yield by improving the action of the cellulolytic enzyme complex. We prospected endo-β-1,4-xylanases from meta-transcriptomes of the termite *Heterotermes tenuis*. In silico structural characterization and functional analysis of an endo-β-1,4-xylanase from a symbiotic protist of *H. tenuis* indicate two active sites and a substrate-binding groove needed for the catalytic activity. No N-glycosylation sites were found. This endo-β-1,4-xylanase was recombinantly expressed in *Pichia pastoris* and *Escherichia coli* cells, presenting a molecular mass of approximately 20 kDa. Enzymatic activity assay using recombinant endo-β-1,4-xylanase was also performed on 1% xylan agar stained with Congo red at 30 °C and 40 °C. The enzyme expressed in both systems was able to hydrolyze the substrate xylan, becoming a promising candidate for further analysis aiming to determine its potential for application in industrial xylan degradation processes.

## 1. Introduction

Bioethanol is a biofuel with the greatest potential to replace oil-based fuels worldwide. The second-generation bioethanol is distinguished from the first-generation by the use of lignocellulosic biomass as raw material in its production. The conversion of lignocellulosic biomass (cellulose, hemicellulose, and lignin) into monomeric sugars is laborious and involves several steps. Enzymatic hydrolysis is one of the most critical steps in this process because of costs and low catalytic efficiency of enzymes at currently used temperatures [1,2]. Therefore, finding new and more efficient enzymes would reduce production costs and increase the efficiency of second-generation bioethanol.

Termites are one of the most efficient lignocellulose decomposers in nature due to an association with symbiont gut microorganisms. This ability has been investigated for several biotechnological applications, especially in biofuel production [3,4]. The association results in a dual enzymatic system comprising endogenous and exogenous carbohydrate-active enzymes (CAZymes) [5,6]. *Heterotermes tenuis* (Isoptera: Rhinotermitidae) is a lower species of termites, presenting a gut microbiota composed of flagellate protists and several species of bacteria and archaea [7]. Since workers partake in the initial breakdown of lignocellulose, their gut microbiotas are even more abundant and diverse than those found in soldier or reproductive castes [8].

Discovering new cellulolytic genes from termite gut microbiota remains a challenge, since part of these microorganisms is non-cultivable. Next-generation sequencing technologies have circumvented such restriction, allowing the complete characterization of microorganism communities in symbiosis with different termites, revealing many diverse genes encoding CAZymes [9]. Here, we considered *H. tenuis* meta-transcriptomes of worker and soldier castes [10] to screen for CAZymes. Glycoside hydrolases (GHs) were the prevalent CAZymes class in both castes, but workers showed a higher diversity of families. The most abundant CAZymes found in the microbiota were three types of cellulases: endoglucanases, cellulose 1,4-β-cellobiosidades, and β-glucosidases.

Cellulases and hemicellulases work coordinately in the enzymatic degradation of lignocellulosic biomass. Since studies commonly investigate cellulases, especially in fungi and bacteria [11], we focused on the hemicellulase endo-β-1,4-xylanase. This enzyme hydrolyzes the β-1,4 bonds of the xylan backbone into xylo-oligomers and D-xylose [12]. The polysaccharide xylan is the main component of hemicellulose, and it contributes to the cohesion and integrity of plant cell walls at the interface between lignin and cellulose [13]. The application of endoxylanases in the bioethanol production chain is relevant because it optimizes the action of cellulases, providing better use of pentoses from the biomass and resulting in higher final sugar production in comparison to the use of cellulases alone [14]. 

In this study, we identified endo-β-1,4-xylanases present in the microbiota of *H. tenuis* and analyzed a protist enzyme for in silico structural characterization, heterologous expression, and functional activity. This newly isolated endo-β-1,4-xylanase belonging to the glycoside hydrolase family 11 (GH11) showed the two active sites necessary for catalytic activity and the substrate-binding groove, but no N-glycosylation site. The recombinant protein has an efficient activity in xylan hydrolysis at two temperatures (30 °C and 40 °C), indicating that this enzyme is a promising candidate to be explored for simultaneous saccharification and fermentation (SSF) processes to increase production of second-generation bioethanol.

## 2. Materials and Methods

### 2.1. Prospection of Cellulolytic Genes in H. tenuis Meta-Transcriptomes

Meta-transcriptomes of *H. tenuis* worker and soldier castes [10] were investigated for the presence of genes encoding carbohydrate-active enzymes (CAZymes), using the meta server dbCAN2 HMMdb v9.0 [15] and the CAZy database [16]. Resulting contigs classified as CAZymes were also annotated with DIAMOND alignment tool [17] against the RefSeq non-redundant (nr) proteins database from NCBI, using the method BLASTp and default parameters except that “-sensitive”, “-salltitles”, “-max-target-seqs 1” and “-max-hsps 1”. Total of CAZy hits for each caste were separated into the following CAZyme classes: Glycoside Hydrolases (GHs), Glycosyl Transferases (GTs), Polysaccharide Lyases (PLs), Carbohydrate Esterases (CEs), Auxiliary Activities (AAs), and the non-catalytic Carbohydrate-Binding Modules (CBMs). Data were plotted separately for termites, protists, bacteria, and fungi, and CAZyme abundance in workers and soldiers was analyzed for statistical significance based on Wilcoxon test (*p* < 0.05).

GH sequences from the microbiota were functionally annotated using the Kyoto Encyclopedia of Genes and Genomes (KEGG) and GhostKOALA [18]. We aimed to identify transcripts annotated according to the following BRITE hierarchical classification: Level 1-Genes and proteins; Level 2-Proteins families: metabolism; Level 3-Enzymes. We considered the database “genus_prokaryotes + family_eukaryotes” for this analysis. Total annotated transcripts for each enzyme were plotted separately for *H. tenuis* workers and soldiers. 

### 2.2. RNA Extraction and cDNA Synthesis

The whole body of 50 worker termites was ground to powder in liquid nitrogen, and total RNA was extracted using the Trizol/chloroform protocol [19]. The integrity was observed by denaturing agarose gel electrophoresis (1.2% agarose, MOPS buffer, and 0.45 M formaldehyde). Samples were quantified and their purity (A260/A280; A260/A230) was evaluated using a NanoVue™ 424 Plus (GE Healthcare, Uppsala, Sweden) spectrophotometer. The RNA was treated with *DNAse* I Amplification Grade (Invitrogen™, Carlsbad, CA, USA), and cDNA synthesis was performed with the *SuperScript™* II RT Kit (Thermo Fisher Scientific, Carlsbad, CA, USA), following the manufacturer’s recommendations. Synthesis efficiency was verified by amplifying a fragment of the endogenous β-actin gene, as previously described [10].

### 2.3. Sanger Sequencing and In Silico Analysis of a Protist Endo-β-1,4-Xylanase

Transcripts encoding the enzyme endo-β-1,4-xylanase (IUBMB Enzyme Nomenclature: EC 3.2.1.8) were identified from the annotations described in Section 2.1. Based on endo-β-1,4-xylanase transcripts comprising complete protein coding sequences (CDS), we designed primers for PCR amplification and sequencing (F: 5′-ATGGTGCTTGCAGCGCTAG-3′ and R: 5′-TCAACTCACCGTCACATCTG-3), using as a template the cDNA sample described in the previous subsection. The PCR reaction was conducted in three replicates using 100 ng cDNA, 10 pmol of each primer, 1× Reaction Buffer, 0.2 mM MgCl_2_, 0.2 mM dNTPs, and 1.25 U Taq Polymerase (Thermo Fisher Scientific, Carlsbad, CA, USA) in a final volume of 25 µL. Water and PCR reagents (but no cDNA) was used as negative control. The PCR was conducted in an initial step at 95 °C for 2 min, followed by 35 cycles of 95 °C for 30 s, 55 °C for 30 s, 72 °C for 1 min, and a final extension step at 72 °C for 5 min. Amplicons were evaluated by electrophoresis in a 1% agarose gel, and sequenced by Sanger sequencing [20] using a facility core.

From the sequence obtained, we conducted in silico predictions regarding the structural features of the encoded protein. We used the InterPro tool [21] to determine the domains, catalytic sites, and motifs composition. Moreover, we performed predictions of glycosylation sites using GlycoEP, considering the standard prediction method and default settings [22]. N-linked glycosylation and O-linked glycosylation sites were investigated based on the Binary Profile of Patterns (BPP) and Composition Profile of Patterns (CPP), respectively, as recommended by the developers. Phosphorylation sites were investigated using NetPhos 3.1, analyzing residues of serine, threonine, and tyrosine [23]. For structural modeling steps, the secondary structure of the protein was inferred by PSIPRED [24] and the tertiary structure inferred by PHYRE2 [25], using the default mode for both software.

### 2.4. Cloning Assays

Molecular cloning was performed according to Sambrook and Russell [26]. PCR assays were performed using two different sets of primers, as described in Section 2.5 and Section 2.6. In both reactions were used 100 ng cDNA, 10 pmol of forward and reverse primers, 1× Reaction Buffer, 0.2 mM MgCl_2_, 0.2 mM dNTPs, and 1.25 U Taq Polymerase (Thermo Fisher Scientific) to a final volume of 50 µL. The amplification conditions were initial denaturation step at 95 °C for 2 min, 35 cycles of 95 °C for 30 s, 68 °C for 30 s, and 72 °C for 1 min, with a final extension step at 72 °C for 5 min. The efficiency of the reaction was confirmed by electrophoresis on a 1% agarose gel. Amplicons were purified using the Agarose Gel Extraction Kit (Cellco, São Paulo, SP, Brazil) and then ligated to pGEM T-Easy propagation vector (Promega, Madison, WI, USA) [27] using T4 DNA ligase, as recommended by the manufacturer.

### 2.5. Construction of pET 28a Vector and Expression in Escherichia coli

The fragment for expression in *E. coli* was amplified using the following primers: F: 5′-CATATGTCGAGTCAGACAGGGACAGACAAC-3′ and R: 5′-GAATTCTTAACTCACCGTCACATCTGAACTCCC-3′. *Nde*I and *EcoR*I restriction sites (respectively underlined) were added to the primers to direct cloning. The signal peptide and the stop codon of the endo-β-1,4-xylanase amplicon were removed. Next, the amplicon was subcloned into the pET28a vector. Both were digested with *Nde* I and *Eco* RI and ligated with T4 DNA ligase according to the manufacturer’s recommendations. Finally, the coding region was cloned into the vector, in-frame with the inducible T7lac promoter, and a 6× HisTag at the N-terminal end.

The recombinant plasmid (named pET28a_HtpXyl) was propagated in *E. coli* DH5α and later used to transform *E. coli Rosetta* (DE3) strains [26]. The transforming colonies were selected on LB medium plates with kanamycin (25 mg/mL) and chloramphenicol (25 mg/mL). Possible recombinant colonies were analyzed by colony PCR (same reaction and cycle used initially). Among the recombinant colonies, one was used to extract the plasmid pET28a_HtpXyl, by using the Fast-n-Easy Plasmid Mini-Prep Kit (Cellco).

For expression assay, recombinant colonies were grown in LB medium containing kanamycin (25 µg/mL) and chloramphenicol (25 µg/mL). Induction was carried out with 0.4 mM IPTG for 16 h, removing aliquots every hour. Cells were resuspended in lysis buffer pH 8.0 and sonicated. The production of the protein was evaluated in polyacrylamide gel, SDS-PAGE 12% [28].

### 2.6. Construction of pPICZαA Vector and Expression in Pichia pastoris

The fragment for expression in *P. pastoris* was amplified using the following primers: F: 5′-AAATATGCGGCCGCTCGAGTCAGACAGGGACAGACAAC-3′ and R: 5′-GCATCTAGAGCACTCACCGTCACATCTGAACTCCC-3′. Next, the *Not* I and *Xba* I restriction sites (respectively underlined) were added to the primers to direct cloning, and the endo-β-1,4-xylanase amplicon was subcloned into the pPICZαA vector. Both were digested with *Not*I and *Xba*I and joined with T4 DNA ligase as per the manufacturer’s recommendations. Finally, the amplicon (as described in the previous subsection, without the signal peptide and stop codon) was cloned into the vector pPICZαA vector in frame with the promoter region of the inducible *AOX1* gene and with a 6× HisTag at the C-terminal end. This system uses the signal peptide present in the vector (α-factor).

The recombinant plasmid pPICZαA_HtpXyl was propagated in *E. coli* DH5α [26]. Colony transformants were selected on Low-Salt (LS) medium plates with zeocin (25 µg/mL). Possible recombinant colonies were analyzed by colony PCR. Among the recombinant positive colonies, one was used to extract the plasmid pPICZαA_HtpXyl, by using the Fast-n-Easy Plasmid Mini-Prep Kit (Cellco).

Approximately 1 µg of the linearized construct pPICZαA_HtpXyl was used to transform the *P. pastoris* X-33 by electroporation (GenePulser, BioRad, Oceanside, CA, USA). The sample was resuspended in 1.0 M sorbitol and incubated for 2 h at 30 °C. Then, it was plated in YPDS medium with increasing concentrations of zeocin (100 µg/mL; 250 µg/mL; 500 µg/mL). Colonies that grew in the 500 µg/mL plate were used for screening in the 24-well plate. Initially, colonies were placed in BMGY medium for 48 h and then induced for 144 h at 30 °C in BMMY medium (25 µg/mL zeocin and 0.75% methanol) at 250 rpm. Next, daily aliquots were collected and methanol was added to the medium to induce the *AOX1* promoter [29]. The supernatant from each aliquot was used for protein expression analysis in 12% SDS-PAGE [28]. Finally, protein-producing colonies were submitted to a new expression, in 100 mL of BMMY medium, within the best expression time analyzed in the screening step.

### 2.7. Qualitative Xylanolytic Activity Assay

The enzymatic activity of the HtpXyl produced in the two expression systems was evaluated on plates containing xylan agar (1% birch xylan; 2% bacteriological agar). For this, 15 µL of protein extract (~5 µg/µL) and serial dilutions (1:2; 1:4; 1:8) were added to each plate. The protein extract expressed in *P. pastoris* was concentrated (10×) due to the low expression observed in the SDS-PAGE as later described. The negative controls for the protein expressed in *E. coli* were LB medium; Rosetta cells (DE3) without plasmid pET28a_HtpXyl (NT), and the sample before induction (NI) with IPTG. The negative controls for the protein expressed in *P. pastoris* were BMMY medium, X-33 cells without plasmid pPICZαA_HtpXyl (NT), and the sample before induction (NI) with methanol. The plates were incubated for 24 h at two different temperatures: 30 °C and 40 °C. After this period, the medium was stained with 0.2% Congo red for 15 min and then washed with 1M NaCl for 15 min. The appearance of lighter zones indicates the presence of xylanolytic activity [30,31]. 

## 3. Results 

### 3.1. CAZy Genes in H. tenuis Meta-Transcriptomes

**Figure 1:**
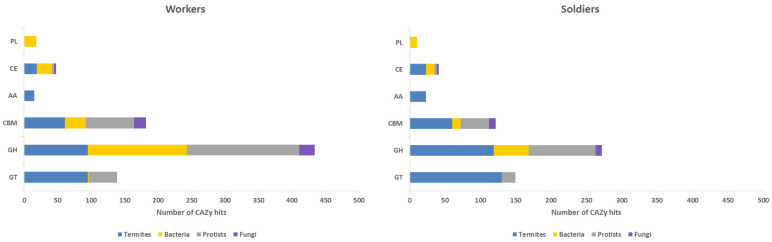
Distribution of CAZy hits across the CAZymes classes found in meta-transcriptomes of *H. tenuis* workers and soldiers in relation to four taxonomic groups: termites, bacteria, protists, and fungi. PL = Polysaccharide Lyases; CE = Carbohydrate Esterases; AA = Auxiliary Activities; CBM = Carbohydrate-Binding Modules; GH = Glycoside Hydrolases; GT = Glycosyl Transferases.

We identified 715 and 542 transcripts annotated as CAZymes in the meta-transcriptomes of *H. tenuis* workers and soldiers, respectively. These numbers correspond to 2.62% (workers) and 2.75% (soldiers) of the total annotated ORFs in these meta-transcriptomes. We also analyzed the number of annotation hits in each CAZyme class (GHs, GTs, PLs, CEs, AAs, and CBMs) for termites, bacteria, protists, and fungi (Figure 1). The number of CAZy hits was 836 for workers and 618 for soldiers. A higher number of termites endogenous CAZys was observed in soldiers, although differences in CAZyme abundance between workers and soldiers were not statistically significant for any taxonomic group considered. 

**Figure 2:**
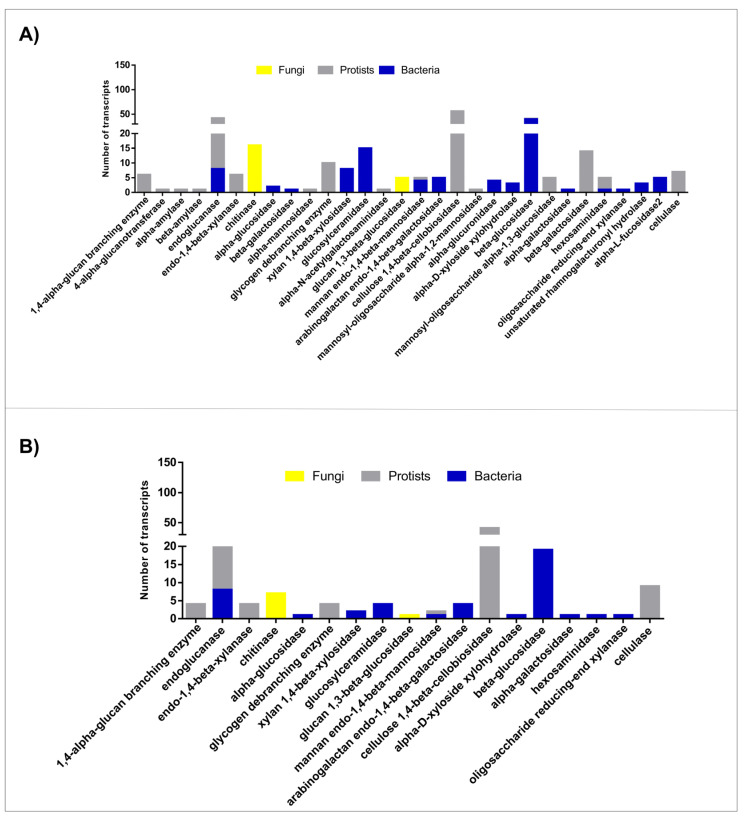
Glycoside hydrolases enzymes from the microbiota of *H. tenuis*, found in meta-transcriptomes of (**A**) workers and (**B**) soldiers.

For both castes, GH was the most abundant class of CAZymes. Our data revealed that 337 out of 715 CAZy worker transcripts (47.1%) belong to 38 GH families (Supplementary Table S1A). Meanwhile, 153 out of 542 CAZy soldier transcripts (28.2%) belong to 23 GH families (Supplementary Table S1B). For both termite castes, transcripts identified as GH members belonging to the microbiota (bacteria, protists, and fungi) were analyzed regarding the Level 3 BRITE hierarchy “Enzymes” (Figure 2).

GH sequences from *H. tenuis* workers’ and soldiers’ microbiota were classified as 30 and 18 different enzymes, respectively. The most abundant glycoside hydrolases were endoglucanases (GH family 5-GH5) and cellulose 1,4-β-cellobiosidades (GH7), which belonged mainly to protists, and β-glucosidases (GH3), which belonged mainly to bacterial species. We also found two types of glycoside hydrolases: glucan 1,3-β-glucosidades (GH55) and chitinases (GH48), from fungi species probably present in the ingested wood or in the soil, as indicated by the two most frequent fungi genera found, *Diaporthe* and *Trichoderma* (Supplementary Table S1).

### 3.2. Endo-β-1,4-Xylanases from Heterotermes tenuis Microbiota

We focused on transcripts encoding xylanases since they are key enzymes during second-generation bioethanol production. We searched for transcripts encoding endo-β-1,4-xylanases (EC 3.2.1.8) and the annotation results showed the presence of six and four different transcripts in the meta-transcriptomes of workers and soldiers, respectively (these transcripts were highlighted in the Supplementary Table S1). Among them, two transcripts in the meta-transcriptome of workers (Work_transcript163 and Work_transcript164) and one in the soldiers (Sold_transcript106) presented complete CDS, all containing 202 amino acid residues. The annotation for these complete transcripts showed high similarity with the protist *Holomastigotoides mirabile*.

### 3.3. Molecular and Protein Structural Features

From the primers designed based on the sequences of the complete transcripts encoding endo-β-1,4-xylanases, we were able to amplify a fragment with the expected molecular weight. This amplicon was sequenced by Sanger method. When compared with the nr/nt NCBI database, it showed a 86.10% of nucleotide similarity with *Holomastigotoides mirabile* CfXyn3-1 mRNA for endo-β-1,4-xylanase (NCBI Accession Number: AB469376.1) [32]. We named the Sanger sequence obtained as “HtpXyl endo-β-1,4-xylanase-like from uncharacterized symbiont protist of *Heterotermes tenuis*” (HtpXyl) (NCBI Accession Number: OL692627). Functional in silico analysis of HtpXyl revealed the GH11 domain and the signatures of the two expected active sites: *GH11_AS_1* (11 amino acids long) and *GH11_AS_2* (12 amino acids long).

**Figure 3:**
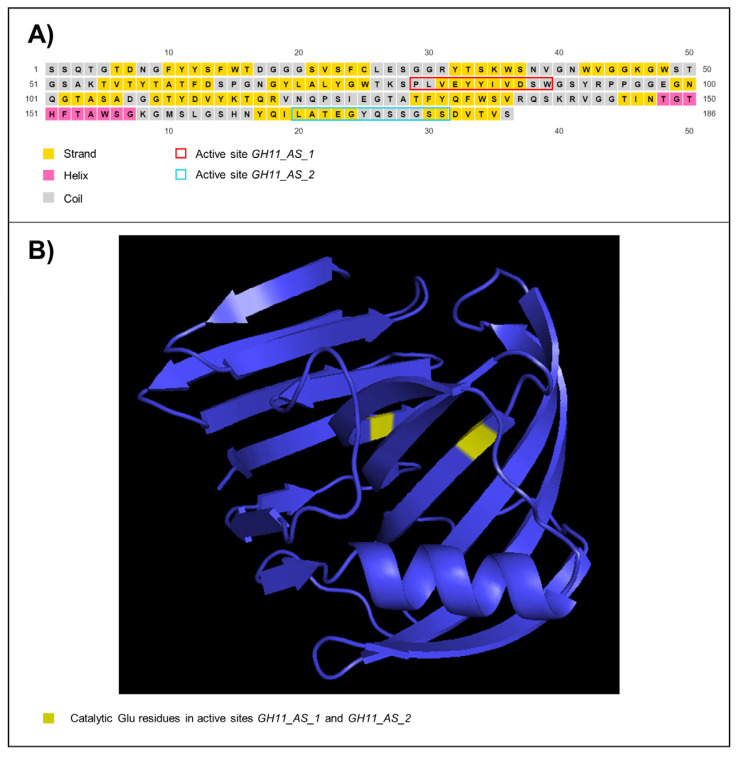
HtpXyl endo-β-1,4-xylanase-like from uncharacterized symbiont protist of *Heterotermes tenuis*. (**A**) Secondary and (**B**) Tertiary structure.

In silico predictions also showed that the encoded protein by HtpXyl presented O-linked glycosylation for 14 out of 47 potential sites, no N-linked glycosylation sites, and 32 phosphorylation sites (Supplementary Figure S1). The inferred secondary structure showed the amount of 15 strands, one helix, and 16 coil structures (Figure 3A). The tertiary structure (Figure 3B) was modeled considering the thermostable mutant of environmentally isolated GH11 xylanase as a template (PDB: 2VUL) [33], with 100% of confidence and 100% of coverage. The structure showed anti-parallel β-strands that bend almost 90° and one helix. Within this structural organization, we observed an open-site structure that allows interaction with the substrate. The two glutamic acid residues essential for catalytic activity [34] are highlighted in the tertiary structure (Figure 3B).

### 3.4. Expression of HtpXyl in Heterologous Systems

**Figure 4:**
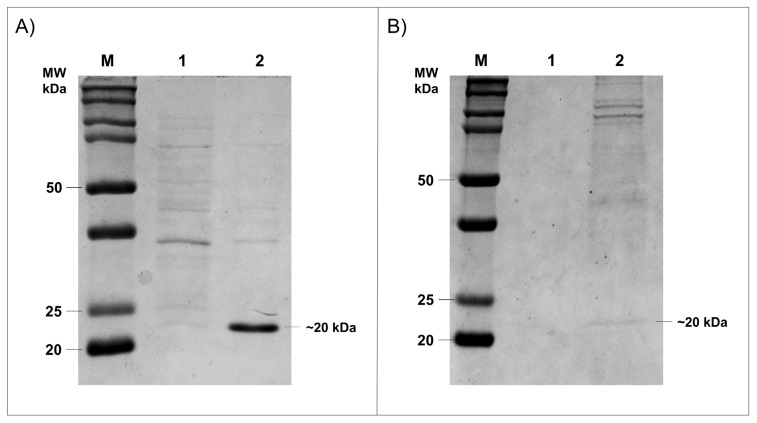
Expression of HtpXyl in bacteria and yeast observed in 12% SDS-PAGE gel. (**A**) HtpXyl expression in *E. coli* Rosetta (DE3). (**1**) the uninduced sample; (**2**) the lysed filtered. (**B**) Expression in *P. pastoris* X-33. (**1**) the uninduced sample; (**2**) the supernatant.

Prokaryotic were the most efficient and robust systems to the heterologous protein expression. However, proteins in which post translation modification is increased could not be efficiently produced in these organisms, therefore a eukaryotic system was necessary. To investigate whether HtpXyl protein has the best enzymatic activity, we expressed it in two different systems. First, the HtpXyl was expressed in *E. coli.* The xylanase was cloned into the pET28a vector and expressed in *E. coli Rosetta* (DE3) cells. Expression of xylanase was analyzed in Coomassie blue stained SDS-PAGE 12%. The analysis revealed the protein HtpXyl expressed with expected molecular masses of 20 kDa (Figure 4A). The solubility of recombinant protein in *E. coli* system was analyzed by the presence of proteins in the supernatant fractions of *E. coli* cells after lysing by sonication. The recombinant xylanase analyzed in SDS-PAGE were present in the soluble fraction of the *E. coli* cells. *E. coli* cells lysate expressing HtpXyl protein was collected, and the obtained extract was used in activity tests.

Since the in silico analysis revealed some glycosylation and phosphorylation sites, we also expressed the HtpXyl protein in the eukaryotic system *P. pastoris*. The xylanase ORF was cloned in the pPICZαA vector and expressed in *P. pastoris* X-33 cells, a strain that does not present mutations in the AOX gene [35,36]. As found in *E. coli* expression, recombinant expression of HtpXyl was achieved in this system. This was observed as a ~20 KDa protein after 24 h of induction with methanol (Figure 4B). After expression in *P. pastoris* system, it was possible to observe the expressed protein in 12% SDS-PAGE gel.

### 3.5. Xylanolytic Activity

HtpXyl activity was evaluated by qualitative assay on plates containing 1% xylan agar. For this purpose, we used xylan agar plates for recombinant crude protein extract (~5 ug of total protein). The formation of halos is related to the affinity of Congo red for carbohydrates, binding to them. Without substrate, the dye does not bind due to the xylanolytic action and is removed in the washing step with 1 M NaCl [31,37].

**Figure 5:**
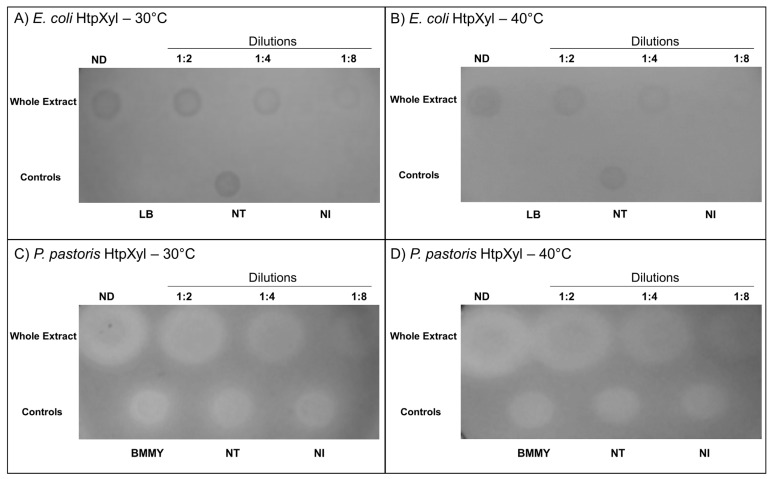
Xylanolytic activity test of HtpXyl in whole protein extract (~5 ug) extracted from *E. coli* and tested at (**A**) 30 °C and (**B**) 40 °C, and from *P. pastoris* at (**C**) 30 °C and (**D**) 40 °C, on 1% birch xylan agar plates stained with 0.2% Congo red. Lighter/whiter zones (halos) indicate xylan degradation. ND = sample non-diluted; NT = without plasmid, non-transformed; and NI = no induction.

Following the incubation of the crude extract in which HtpXyl was expressed and after Congo red treatment, it was possible to see the formation of lighter zones (halos) in both *E. coli* and *P. pastoris* at both tested temperatures (Figure 5). This indicates that HtpXyl was expressed in its active form, degrading the substrate efficiently in the two tested systems. In addition to the apparent increase in the expression of *E. coli*, and the xylanolitic activity observed in the *P. pastoris* control crude extract, yeast expression showed a consistent increase in the degradation of xylan activity in all dilutions tested and at both temperatures (Figure 5C,D). This may be due to post-translational modifications carried out by this eukaryotic system. 

The presence of halos in non-transformed (NT) and non-induced (NI) controls may have been caused by the action of endogenous hydrolases secreted by *P. pastoris* X-33 cells or components present in the cell lysate of *E. coli* Rosetta DE3 cells. The formation of halos in the control containing only the culture medium (BMMY) may have occurred due to the indirect action of compounds present in this complex culture medium (yeast extract; peptone; 1.0M phosphate buffer, pH 6.0; YNB 10×; biotin 0.02% and absolute methanol), such as yeast extract [36,38]. In both cases, we can observe that the halos formed are smaller and with more indistinct margins than the degradation halos in which there is the recombinant protein, demonstrating the direct action of HtpXyl in the degradation of xylan.

## 4. Discussion

### 4.1. CAZymes from Heterotermes tenuis Microbiota and Selection of an Endo-β-1,4-Xylanase

We investigated the presence of cellulolytic genes in the meta-transcriptomes of *H. tenuis* workers and soldiers and found that 2.62% (in workers) and 2.75% (in soldiers) of the ORFs reported by Campanini and colleagues [10] consisted of them. In general, cellulolytic genes account for 1–3% of the proteins encoded by most organisms [39]. The total number of CAZy hits was higher than the number of transcripts annotated. This result is due to the annotation of a catalytic CAZyme and a non-catalytic carbohydrate-binding module (CBM) in the same given transcript. The association between CBMs and CAZymes is essential to promote bonding with the substrate [40].

Soldiers have more endogenous CAZys than workers according to our results. Although termite enzymes may contribute to cellulose digestion, most CAZymes from termites cannot fully break down the lignocellulosic components [4,41]. Therefore, the association with microbial gut symbionts is necessary for an efficient lignocellulose decomposition [42]. Workers are responsible for foraging and nest feeding. Since they are responsible for the initial digestion of lignocellulose, they are expected to present a higher number of endosymbionts in comparison to soldiers or reproductive castes [8,43]. This pattern was indeed observed regarding the presence of CAZymes in *H. tenuis* meta-transcriptomes, since our results indicated that hits with bacteria and protists were higher in workers for all enzyme classes. Differences in CAZyme abundance between workers and soldiers, considering absolute numbers of hits for each taxonomic group (termites, bacteria, protists, and fungi), were not statistically significant. However, focusing on the GH class we observed a considerable diversity between workers and soldiers regarding both the number of GH families and the number of different GH enzymes from their microbiota, as we discussed below. It suggests that the heterogeneity of the CAZymes datasets, when analyzed globally, may have skewed the statistical results.

The GH class was the most abundant class of CAZymes for both termite castes studied, especially considering the microbiota. The workers’ microbiota showed a higher diversity of GH families when compared with soldiers as expected. KEGG BRITE annotation also indicated a higher diversity in the workers’ microbiota as well as a significant number of glycoside hydrolases. The most abundant enzymes were endoglucanases, cellulose 1,4-β-cellobiosidade, and β-glucosidase. These enzymes act synergistically in the cellulase system in three steps: (i) first, endonucleases cleave the linear chains of glucose; (ii) the exposed ends of the substrate become available for the activity of exoglucanases (such as cellulose 1,4-β-cellobiosidades); and (iii) β-glucosidases complete the degradation of cellulose [44]. Our results suggest that the second step is performed exclusively by protists enzymes within *H. tenuis* microbiota, while bacterial enzymes are responsible for the third step of the process.

Since cellulases have been widely studied, we focused on the hemicellulase endo-β-1,4-xylanase. This enzyme hydrolyzes the polysaccharide xylan, the main component of hemicellulose. All endo-β-1,4-xylanase transcripts identified in the meta-transcriptomes were annotated as belonging to termite gut symbiotic protists. No endogenous termite endo-β-1,4-xylanase genes were detected. Termites with gut symbiotic flagellates removed lost the xylanolytic activity, indicating that termites lack this enzyme [45]. Our results corroborate this hypothesis.

### 4.2. HtpXyl Features

HtpXyl presented the GH11 domain, indicating that it belongs to the GH11 family. It also presents two expected active sites signatures, indicating both active sites needed for endo-β-1,4-xylanase activity. These active sites are conserved and centered on a glutamic acid residue. While one of them acts as a general acid/base catalyst, the other one acts as a nucleophile [34].

We identified in HtpXyl O-linked glycosylation sites and phosphorylation sites, but no N-linked glycosylation sites. Previous studies have reported one to four sites of N-linked glycosylation in xylanases from fungi and bacteria [46,47,48], reporting the importance of the presence of N-linked glycosylation sites for xylanases activity. Nonetheless, HtpXyl was able to degrade its substrate without needing N-linked glycosylation sites. We have not found any studies investigating N-linked glycosylations in protist xylanases. Therefore, we hypothesize that protist xylanases do not need N-glycosylation sites to be functional. Further studies are needed to evaluate this hypothesis.

The bonds of the xylan backbone that endo-β-1,4-xylanases hydrolyze are not randomly chosen, but based on the analysis of branches, chain length, and presence of substituents on the substrate molecule [49,50,51]. The efficient catalytic activity of these enzymes is thus closely related to how the structure of their substrate-binding groove are folded. Our predictions of the HtpXyl tertiary structure indicate that it folds into a “jelly-roll shape”, showing a substrate-binding groove. Both characteristics have been described for enzymes of the GH11 family [52]. We also found the two catalytic glutamic acid residues at opposite sides of the groove, a condition that is needed for hydrolysis reaction [33]. The results of the in silico analyzes allowed to conclude that HtpXyl had the necessary conditions for catalytic activity.

### 4.3. HtpXyl Expression Profiles and Enzyme Activity

Heterologous protein expression is one of the first steps to test enzymes for biotechnological and industrial applications. Here, we used two different expression systems to produce recombinant xylanases. Although *E. coli* is the main recombinant protein expression used in the literature, prokaryotic systems do not perform essential post-translational modifications possibly resulting in the lack or decrease of protein activity [53,54]. This limitation can be circumvented by expressing the protein of interest in a eukaryotic organism system such as *P. pastoris.* This organism can carry out different post-translational modifications, such as glycosylation, methylation, and proteolytic processing [55]. In addition, due to the α-factor, the recombinant protein expressed in *P. pastoris* is secreted into the extracellular medium. Depending on the application, the whole secreted crude extract could be used for biotechnological purposes, reducing the costs of the purification process.

The recombinant HtpXyl enzyme expressed in both systems (as shown in Figure 5) was able to degrade the xylan substrate without purification at two different temperatures (30 °C and 40 °C). The enzymatic step of second-generation bioethanol production process usually takes place at high temperatures (50 °C to 60 °C). As the temperature needs to be decreased to approximately 30 °C for the fermentation step, it generates an increase in production costs and use of water [56]. Thus, for bioethanol production, enzymes used in the enzymatic step should ideally act at temperatures close to those used for yeast fermentation. Another alternative would be to prospect for enzymes that act in a range of 40 °C so that they can be used in association with thermotolerant yeasts [57,58,59]. Our qualitative xylanolytic activity assays indicated that HtpXyl has the potential to be efficient in these two described situations.

The discovery/development of new more active and temperature stable enzymes is paramount to making second-generation bioethanol production economically viable at an industrial scale. We presented an initial characterization of HtpXyl, a new endo-β-1,4-xylanase from a symbiotic protist of the termite *H. tenuis.* Our results show that HtpXyl is a promising candidate to be explored in further studies aiming its use in simultaneous saccharification and fermentation (SSF) processes, at regular or higher temperatures in association with thermotolerant yeasts, which have the potential to reduce second-generation production costs.

## 5. Conclusions

The prospection of CAZymes from meta-transcriptomes of workers and soldiers of *H. tenuis* indicated that GHs were the most abundant CAZymes in both castes, with a great number of enzymes belonging to bacteria and protists, especially in the worker caste. Moreover, worker caste showed a higher diversity regarding both the number of different GH families and the number of different enzymes from the microbiota. We described a new endo-β-1,4-xylanase from a symbiotic protist of *H. tenuis*. This enzyme, called HtpXyl, has the two active sites and a substrate-binding groove necessary for catalytic activity. We expressed HtpXyl in *P. pastoris* and *E. coli* and found that the recombinant proteins were able to hydrolyze the substrate xylan when expressed in both systems. Based on the findings of this study, we conclude that HtpXyl is a potential candidate for further biochemical and functional analysis aiming its application in biotechnological processes of xylan degradation, including second-generation bioethanol production.

## Data Availability

Nucleotide and amino acid sequences of the endo-β-1,4-xylanase described here were submitted to the GenBank database under accession number OL692627.

## Supplementary Materials

The following supporting information can be downloaded at: https://www.mdpi.com/article/10.3390/microorganisms10050906/s1, Table S1: Transcripts from *Heterotermes tenuis* meta-transcriptomes of (A) workers and (B) soldiers, identified as microorganisms CAZymes; Figure S1: Post-translational modifications identified in HtpXyl. (A) O-linked glycosylated sites. Red colored and underlined amino acids are potentially O-linked glycosylated. (B) Phosphorylation sites. Colored lines passing through the threshold indicate sites potentially phosphorylated.
