# Supplementary material for: Identification of a New Endo-β-1,4-xylanase Prospected from the Microbiota of the Termite Heterotermes tenuis
Source: Microorganisms. 2022 Apr 26;10(5):906. doi: 10.3390/microorganisms10050906 (PMC9143652; doi:10.3390/microorganisms10050906)
Supplement: Supplementary file 1 [file microorganisms-10-00906-s001.zip › microorganisms-1547244-supplementary/microorganisms-1547244 for proof suppl/Suppl.Figure S1.pdf]

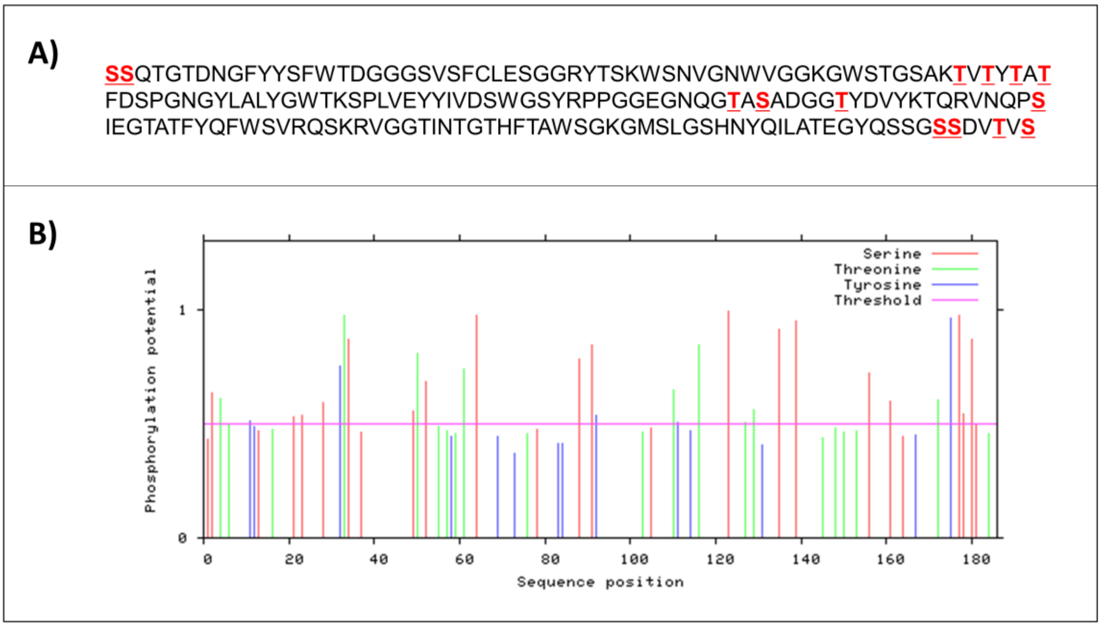

Figure S1: Post-translational modifications identified in HtpXyl. (**A**) O-linked glycosylated sites. Red colored and underlined amino acids are potentially O-linked glycosylated. (**B**) Phosphorylation sites. Colored lines passing through the threshold indicate sites potentially phosphorylated.
